# Synthesis and Antimicrobial Activity of New Mannich Bases with Piperazine Moiety

**DOI:** 10.3390/molecules28145562

**Published:** 2023-07-21

**Authors:** Sara Janowska, Sylwia Andrzejczuk, Piotr Gawryś, Monika Wujec

**Affiliations:** 1 Department of Organic Chemistry, Faculty of Pharmacy, Medical University of Lublin, 4a Chodzki Street, 20-093 Lublin, Poland; 2 Department of Pharmaceutical Microbiology, Faculty of Pharmacy, Medical University of Lublin, 1 Chodzki Street, 20-093 Lublin, Poland; sylwia.andrzejczuk@umlub.pl; 3 Students Research Group, Department of Organic Chemistry, 4a Chodzki Street, 20-093 Lublin, Poland; pgawrys671@gmail.com

**Keywords:** piperazine derivatives, Mannich reaction, antimicrobial activity, antifungal activity

## Abstract

A series of novel Mannich bases were designed, synthesized, and screened for their antimicrobial activity. The target compounds were synthesized from 4-(3-chlorophenyl)-5-(3-fluorophenyl)-2,4-dihydro-3*H*-1,2,4-triazole-3-thione and different piperazine derivatives. The structures of the products were confirmed by ^1^H and ^13^C NMR and elemental analysis. The activity of piperazine derivatives against bacteria (Gram-positive: *Staphylococcus epidermidis*, *Staphylococcus aureus*, *Micrococcus luteus*, *Bacillus cereus*, and *Bacillus subtilis*; Gram-negative: *Escherichia coli*, *Pseudomonas aeruginosa*, *Klebsiella pneumoniae*, and *Proteus mirabilis*) and yeasts (*Candida glabrata*, *Candida krusei*, and *Candida parapsilosis*) was determined by the minimum inhibitory concentration and minimum bactericidal concentration values. Significant activity was observed against Gram-positive bacteria, mainly staphylococci (**PG7**–**PG8**) and bacteria of the genes of *Micrococcus* and *Bacillus* (**PG1-3**), as well as selected strains of Gram-negative bacteria, including bacteria of the *Enterobacteriaceae* family (**PG7**), while all tested compounds showed high fungistatic activity against *Candida* spp. yeasts, especially *C. parapsilosis*, with MICs ranging from 0.49 µg/mL (**PG7**) to 0.98 µg/mL (**PG8**) and 62.5 µg/mL (**PG1-3**). In conclusion, the results obtained confirm the multidirectional antimicrobial activity of the newly synthesized piperazine derivatives. Furthermore, in silico studies suggest that the tested compounds are likely to have good oral bioavailability. The results obtained will provide valuable data for further research into this interesting group of compounds. The library of compounds obtained is still the subject of pharmacological research aimed at finding new interesting biologically active compounds.

## 1. Introduction

The treatment of infectious diseases remains an important and challenging issue due to the accumulation of many problems related to therapy, such as the development of resistance of pathogens to currently known drugs and the very rapid increase in the number of opportunistic fungal infections in immunocompromised patients. This group includes cancer patients treated with methods such as chemotherapy and radiotherapy, as well as people taking immunosuppressive drugs [1,2,3]. The rapidly increasing resistance of microorganisms, both bacteria and fungi, to available treatments is a global medical problem of high priority. Infections of patients in hospitals and closed care facilities are the most conducive to the development of drug resistance in pathogens [4]. The microorganisms that evolve in the hospital environment have become resistant to the most effective drugs. Every day, patients worldwide are diagnosed with infections caused by resistant strains of bacteria and fungi [4]. Because the rate of emergence of new multi-resistant, hospital-acquired strains is faster than the rate at which new drugs are discovered and brought into medical use, the shortage of effective antimicrobials, including antifungals, is now a global problem [5].

The overall objective, as defined by international institutions, is to slow down the development of antimicrobial resistance and to preserve the efficacy of currently available and used antibiotics and/or chemotherapeutics in the control of animal and human diseases. The aim is not only to reduce the volume of antimicrobial sales but also to place these products under strict surveillance to promote good antimicrobial stewardship practices by optimizing the use of these products and limiting their use to cases where it is necessary for the treatment, control, or prevention of infectious disease. The antimicrobial resistance situation for bacterial species reported to the AMR surveillance networks by bacterial species, antimicrobial group, and geographical region was provided by the European Centre for Disease Prevention and Control (ECDC) [6].

Opportunistic microorganisms, including *Candida* fungi, pose a serious threat. A major clinical problem is that of low pathogenicity pathogens with high resistance rates to known antimicrobial agents. Such yeasts in the patient’s body can gradually become resistant to subsequent drugs without causing disease symptoms, while at the same time posing a serious threat to people with reduced immunity [7]. This includes those with health conditions that favor opportunistic infections, comprising AIDS, diabetes, long-term antibiotic therapy, and the use of immunosuppressive drugs and corticosteroids. Patients most at risk of invasive systemic candidiasis include low-birth-weight babies, people recovering from surgery, patients in intensive care units and those with a compromised immune system [7]. Patients at the greatest risk of developing *Candida*-associated infections due to their health status or age group are in the hospital environment, and hospitals are the places where multidrug-resistant strains are developing most rapidly [8].

Studies have shown that the two position in the triazole heterocyclic ring is the target of many reactions, leading to the obtaining of biologically active compounds [9,10,11]. One of the reactions that can be used to obtain 1,2,4-triazole derivatives with potential biological activity is the Mannich reaction [12]. By using the Mannich reaction, it is possible to carry out many modifications to the chemical structure. There are many studies showing the antibacterial and antifungal effects of Mannich bases obtained by this reaction [4,13,14,15,16]. The aminomethylation reaction, which is the Mannich reaction, can also be used to increase the bioavailability of the molecule. Aminomethylation has a positive effect on the hydrophilic properties of drugs by introducing a polar element into their structure [17].

The resulting Mannich bases may contain various types of heterocyclic systems in their structure. Thanks to this, they are an interesting object of further chemical modifications, which can lead to obtaining new derivatives with potential biological activity [17]. The Mannich reaction enables the introduction of an amine fragment into various chemical structures, which makes it possible to increase the affinity of heterocyclic molecules to the appropriate molecular target. At the same time, 1,2,4-triazole derivatives, which are known for their antibacterial and antifungal activity [18,19,20], can be successfully used as substrates in the Mannich reaction [21]. The choice of piperazine derivatives as one of the substrates used in the Mannich reaction was associated with the positive effect of the presence of this group on the antimicrobial activity of other groups of drugs [22]. The structural element, which in this case is the piperazine group, played an important role in research on improving the potency of antibacterial drugs [23]. An example of a drug containing piperazine in its structure is ciprofloxacin, which is a fluoroquinolone antibiotic with a mechanism of action based on the inhibition of the bacterial enzyme DNA gyrase [23]. Our literature review of studies on the activity of piperazine derivatives indicates that many compounds from this group have antibacterial [24,25,26,27,28] and antifungal activity [29].

All of the above considerations led us to synthesize a series of new Mannich bases that are derivatives of 1,2,4-triazole. Due to the fact that the introduction of a halogen atom into an organic molecule significantly affects its physical and chemical properties [30,31], and one of the well-known antibacterial compounds is fluoroquinolone obtained by introducing fluorine into the quinolone structure, we planned the synthesis of triazole derivatives containing fluorophenyl and chlorophenyl fragments. We designed a series of compounds as potential drugs with antifungal and antibacterial properties, intended for use against drug-resistant strains of bacteria and fungi. The structures of the compounds obtained were confirmed by ^1^H NMR and ^13^C NMR spectroscopy. The antimicrobial activity of the compounds obtained was determined using the microdilution broth method, with the MIC (minimal inhibitory concentration) and MBC (minimal bactericidal concentration) values performed against a wide spectrum of reference strains of bacteria and fungi.

## 2. Results and Discussion

### 2.1. Chemistry

**Scheme 1:**
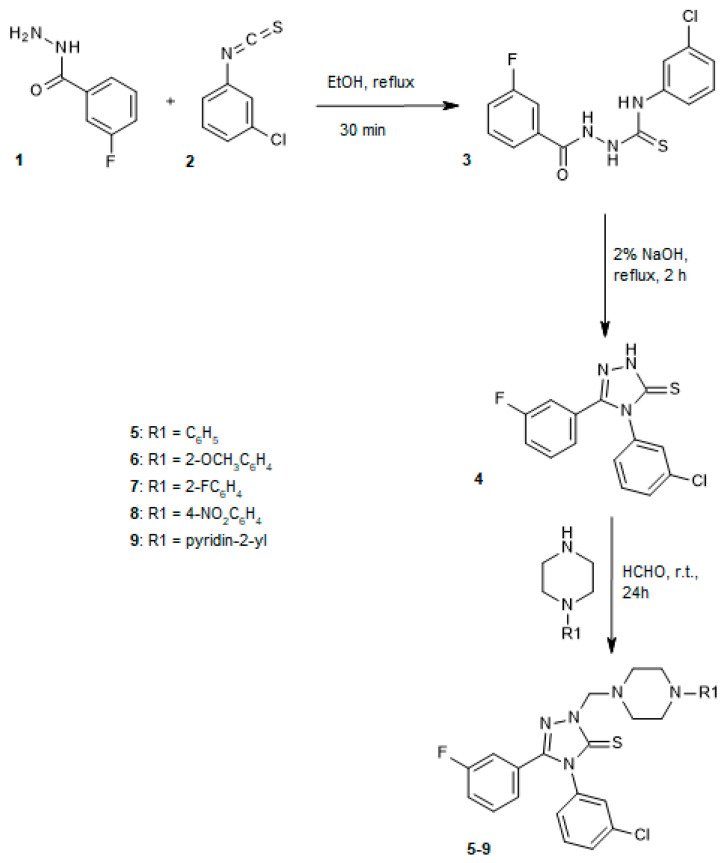
Synthetic route of new Mannich bases.

The synthesis of the new Mannich base compounds was carried out according to the synthetic route shown in Scheme 1.

In the first step of the reaction, 3-chlorophenyl isothiocyanate (**2**) was added to the 3-fluorobenzoic acid hydrazide (**1**) dissolved in anhydrous ethanol. The reaction was carried out by heating the reactants under reflux for half an hour. In this way, 4-(3-chlorophenyl)-1-(3-fluorobenzoyl)thiosemicarbazide (**3**), which is a substrate for all further reactions, was obtained with a high yield (96%). The physicochemical data of the synthesized compound were consistent with the literature data [32]. According to the research results, the method used by us is faster and more efficient than the method based on solvent-free synthesis [33,34]. Then, the alkaline cyclization of the previously prepared thiosemicarbazide (**3**) was carried out using a 2% sodium hydroxide solution. In this way, 4-(3-chlorophenyl)-5-(3-fluorophenyl)-2,4-dihydro-3*H*-1,2,4-triazole-3-thione (**4**) was synthesized with physicochemical data consistent with the literature data [32].

Mannich bases (**5**–**9**) were prepared by reactions of 4-(3-chlorophenyl)-5-(3-fluorophenyl)-2,4-dihydro-3*H*-1,2,4-triazole-3-thione with various piperazines and formaldehyde (37%) in ethanol (96%). The reaction mixture was left for 24 h at room temperature, thereby leading to the spontaneous precipitation of the reaction product.

We planned to use different piperazine derivatives containing electron-donating and electron-withdrawing substituents in the *ortho* and *para* positions of the phenyl ring. The conducted research is preliminary. Such a selection of substituents was intended to show the influence of the nature of the substituent, as well as its position on microbiological activity. In addition, we used piperazine, which has a pyridine or furan system in place of the phenyl ring, to determine the effect of the substituent at the four position of piperazine on activity.

The efficiency of the processes carried out was in the range of 32–62%. During the research, four of the planned compounds failed to be obtained, despite changing the reaction conditions, consisting in heating the reaction mixture, changing the solvent, or extending the reaction time.

The structures of the obtained compounds were confirmed using proton and carbon magnetic resonance spectroscopy (^1^H NMR). The course of the aminomethylation reaction is evidenced by the lack of a signal for the proton at the N2 nitrogen atom of the 1,2,4-triazole ring. Moreover, in the ^1^H NMR spectra in the range of 5.25–5.28 ppm, there is a singlet corresponding to the methylene group connecting the triazole ring with the piperazine ring. The signal of eight piperazine aliphatic protons was visible as two multiplets in the range of 2.92–3.54 ppm. In the ^13^C NMR spectra of titled compounds (**5**–**9**), aliphatic carbons were visible in the range 43.59–69.71 ppm (Supplementary Materials Figures S1–S10).

The compounds from the group of Mannich bases mentioned above are new molecules not previously described in the scientific literature (the details are in the Materials and Methods section). The assessment of antimicrobial activity described later in the article was also carried out for the first time.

### 2.2. Antimicrobial Activity

In the search for effective antimicrobial agents, the literature provides information on a number of heterocyclic compounds, including piperazine derivatives, which have shown a broad spectrum of pharmacological effects, including antibacterial and antifungal activity. Medicinal chemists have had great success in modifying the basic chemical structures of known antibiotics, both natural and synthetic, in which the heterocyclic nucleus forms part of the pharmacophore necessary for specific pharmacological activity. Piperazine is a medically important heterocyclic nucleus consisting of a six-membered ring containing two nitrogen atoms in opposite positions in the ring. The piperazine nucleus has been classified as privileged and is frequently found in biologically active compounds in many different therapeutic areas [35].

Piperazine-containing ciprofloxacin dimers are often described as potent antimicrobial agents with high activity against resistant or multidrug-resistant strains [4,18,19,21,32,33,34,36]. In this study, all new piperazine derivatives obtained were tested against a broad panel of reference strains of either Gram-positive and Gram-negative bacteria or yeasts were carried out at the Department of Pharmaceutical Microbiology at the Medical University of Lublin, Poland. The susceptibility of selected pathogenic microorganisms to PG compounds, as indicated by their MIC and MBC values, is presented in Table 1.

**Figure 1:**
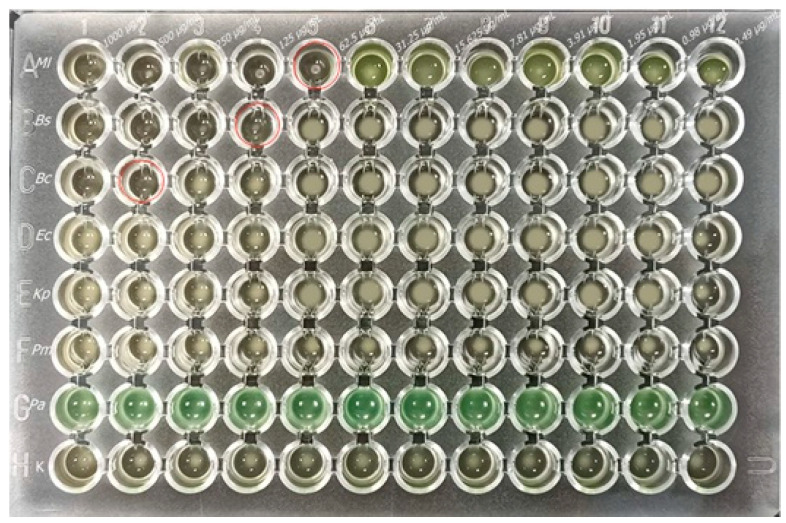
Minimum inhibitory concentration plate for compound **5** on 96-well plate. **Abbreviations:** Ml—*Micrococcus luteus* ATCC 10240, Bs—*Bacillus subtilis* ATCC 6633, Bc—*Bacillus cereus* ATCC 10876, Ec—*Escherichia coli* ATCC 25922, Kp—*Klebsiella pneumoniae* ATCC 13883, Pm—*Proteus mirabilis* ATCC 12453, Pa—*Pseudomonas aeruginosa* ATCC 27853, K—PG1 compound control.

The piperazine derivatives (**5**–**7**) were the most active against *M. luteus*, *B. subtilis*, and *B. cereus*, with MIC values ranging from 125 to 500 µg/mL for **5**, **7**, and 1000 mg/mL for **6**, and MBC values ranging from 125 to 1000 µg/mL. They were also bactericidal against these strains of Gram-positive bacteria, with the exception of compound **6**, which was bacteriostatic (Figure 1). None of these three compounds showed activity against any strain of Gram-negative bacteria.

**Figure 2:**
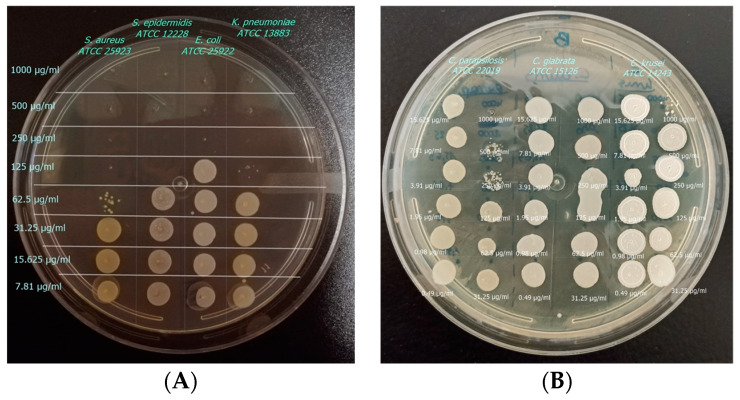
Minimum bactericidal concentration assay on (**A**) Mueller–Hinton agar medium obtained for **8** and (**B**) on Mueller–Hinton agar + 2% glucose medium obtained for compound **9**.

The compound **8** also demonstrated promising antimicrobial activity against the following strains of either Gram-positive (staphylococci and *B. cereus*) or Gram-negative (*E. coli* and *K. pneumoniae*) bacteria with MIC values of 125–500 µg/mL. A ratio of MBC to MIC < 4 demonstrating the bactericidal effect of the compound **8** was shown against *S. aureus*, *B. cereus*, and *Enterobacteriaceae* rods, including *E. coli* and *K. pneumoniae* (Figure 2). Chemically, it is characterized by the presence of a 4-nitrophenyl moiety attached to the piperazine ring. The compound **9** presented favorable activity against Gram-positive bacteria, including staphylococci (MIC = 250 µg/mL) and *B. subtilis* (MIC = 125 µg/mL). This piperazine derivative was found to be bacteriostatic (MBC/MIC = 2).

The problem of resistance to third-generation cephalosporins and carbapenems, which is higher in *Klebsiella pneumoniae* than in *Escherichia coli*, has been highlighted, followed by carbapenem resistance, common in *Pseudomonas aeruginosa* and *Acinetobacter* species and at a higher percentage than in *K. pneumoniae*. A global resistance gradient was observed, with higher rates in the European region [6]. Based on the data collected, it can be assumed that the high bacteriostatic, bactericidal, and fungistatic activity is related to the presence of a nitro group attached to the aromatic system. The presence of a nitro group in the structure of the molecule is often associated with antimicrobial activity and, unfortunately, also with its toxic effect. It is therefore advisable to conduct further studies to investigate the cytotoxicity of **8**, which is going to be studied in more detail.

The newly synthesized compounds were evaluated for their antifungal activity against *Candida* spp. reference strains (*C. glabrata*, *C. parapsilosis*, and C. *krusei*). The microdilution method was used at concentrations of 0.49–1000 μg/mL, while the positive control was posaconazole. After incubation of the plates, the activity of the compounds was assessed as the minimum inhibitory concentration (MIC) that visibly inhibited the growth of the fungi. All newly synthesized piperazine derivatives showed significant fungistatic activity against only one of three *Candida* spp. strains—*C. parapsilosis* ATCC 22019, with MICs ranging from 0.49 and 0.98 µg/mL (shown by **8** and **9**, respectively) to 62.5 µg/mL shown by compounds **5**–**7**, regardless of the types of substituents present in the structure of thiosemicarbazide.

*C. parapsilosis* is the second most common *Candida* spp. species generally isolated from blood cultures in many countries [37,38]. The prevalence of *C. parapsilosis* in the hospital environment is related to the fact that this opportunistic species has a strong ability to form and grow in biofilm structure that can adhere to biotic and abiotic surfaces (e.g., medical devices with vascular, orthopedic, and other implants and vascular and urinary catheters). This dramatically increases the patient’s risk of developing a difficult-to-treat blood infection. In this context, all of the newly synthesized piperazine derivatives, which are active against *C. parapsilosis* at low drug concentrations ranging from 0.49 to 62.5 µg/mL, become promising materials for further research. The MFC/MIC ratios indicated that the new piperazine derivatives had static activity against yeast. On the basis of the results obtained, it can be concluded that the tested substances do not have fungicidal properties.

We also found that in the series of molecules discussed, the type of substituent attached to the pyridine ring affects the antimicrobial activity. Within this group of compounds, the nitrophenyl substituent in the four position of piperazine proved to be the most advantageous, promoting both antibacterial and antifungal activity.

Significant activity of some of the newly synthesized piperazine derivatives was observed against Gram-positive bacteria, mainly staphylococci (**8**–**9**) and bacteria of the genus *Micrococcus* and *Bacillus* (**5**–**7**), as well as selected strains of Gram-negative bacteria, including *Enterobacteriaceae* (**8**). All tested compounds showed high fungistatic activity (MFC/MIC ratio > 4) against *Candida* spp. yeasts, only against *C. parapsilosis*, at MICs ranging from 0.49 µg/mL (**8**) to 0.98 µg/mL (**9**) and 62.5 µg/mL (**5**–**7**). The results obtained in recent years by other authors indicate that the compounds obtained by us are characterized by better antifungal and antibacterial activity. Only ciprofloxacin hydrides turned out to be definitely more active [23].

In conclusion, the results obtained confirm the multidirectional antimicrobial activity of the newly synthesized piperazine derivatives and show how changing the substituents in their structure can affect the activity of this class of compounds against different microorganisms. The results obtained will provide valuable data for further research into this interesting group of compounds. The library of compounds obtained is still the subject of pharmacological research aimed at finding new interesting biologically active compounds.

### 2.3. In Silico Pharmacokinetic Prediction

All derivatives were predicted for possible pharmacokinetic and drug-likeness properties using the SWISSADME server (http://www.swissadme.ch/index.php, accessed on 17 June 2023).

The obtained simulated results concerning the physicochemical properties of compounds in the context of the Lipiński rule. Four compounds meet the rule of five (Ro5) [39], suggesting that they are likely to have good oral bioavailability and membrane permeability. The results confirming this statement are presented in Table 2.

Based on the obtained results, it can be predicted that the tested molecules will be largely absorbed through the gastrointestinal tract, without compounds with a nitro group (Table 3).

However, only one of them without substituent in the phenyl ring in position four of piperazine should cross the blood–brain barrier. This means that while the other four compounds may be well absorbed by the body, they may not have a significant effect on the central nervous system. In addition to the above findings, the water solubility of the compounds has been found to be rather poor. Finally, it was calculated that the compounds not only obey Lipinski’s rule of five, but also satisfy the descriptors of Veber’s rule (Table 4) [40]. Veber’s rule relates to the number of rotational bonds and the polar surface area of a compound, which can affect its oral bioavailability and pharmacokinetics.

In conclusion, based on the presented results, the tested compounds are expected to have good oral bioavailability and membrane permeability, with general high absorption in the gastrointestinal tract. However, they may have limited water solubility and are not expected to cross the blood–brain barrier.

## 3. Materials and Methods

### 3.1. Chemistry

In order to check the reaction course, the thin-layer chromatography method was used with Merck chromatographic plates—aluminum oxide 60F-254. The mobile phase was a mixture of ethanol and chloroform in a volume ratio of 3:7. Then, after drying the chromatograms, they were developed by detection with a UV lamp with a wavelength of λ = 254 nm. The melting points were determined using the Böetius apparatus. The structures of the new compounds were confirmed based on the nuclear magnetic resonance (^1^H NMR) spectra performed with the Brucker Avance 600 MHz spectrometer. DMSO-*d*_6_ was used as the solvent. The chromatographic measurements were performed using an LC/MS system consisting of UHPLC chromatograph (UltiMate 3000, Dionex, Sunnyvale, CA, USA) connected with the linear trap Quadrupole-Orbitrap mass spectrometer (LTQ-Orbitrap Velos from Thermo Fisher Scientific, San Jose, CA, USA) equipped with ESI source. In all the analysis, a Gemini C18 column (4.6 × 100 mm, 3 µm) (Phenomenex, Torrance, CA, USA) was used for chromatographic separation. During the chromatographic process, isocratic elution was used. Mobile phase A (25%) was 25 mM ammonium formate in water; mobile phase B (75%) was 25 mM ammonium formate in acetonitrile. The mobile phase flow rate was 0.5 mL/min by 30 min in each analysis. In the course of each run, MS spectra in the range of 200–600 *m*/*z* were collected continuously. The ESI was operated in positive polarity modes under the following specific conditions: spray voltage −4.5 kV; sheath gas −35 arbitrary units; auxiliary gas −5 arbitrary units; sweep gas −5 arbitrary units; and capillary temperature −300 °C. Nitrogen (>99.98%) was employed as sheath, auxiliary, and sweep gas. The scan cycle used a full-scan event at the resolution of 140,000.

The 4-(3-chlorophenyl)-5-(3-fluorophenyl)-2,4-dihydro-3*H*-1,2,4-triazole-3-thione (**4**) was prepared following a reported procedure [13].

**Synthesis of Mannich bases.**

A total of 0.2 g of the 1,2,4-triazole-3-thione derivative (**4**) was dissolved in 10 mL of 96% ethanol. Then a molar amount of appropriate amine and 5 drops of 40% formaldehyde solution were added and left for 24 h at room temperature. The progress of the reaction by TLC chromatography was checked. After confirming that no substrate appeared in the sample, the resulting precipitate was filtered and dried in the air. The final compounds obtained were purified by crystallization from 96% ethanol. The following amines for the reaction were used: 1-phenylpiperazine, 1-(2-methoxyphenyl)piperazine, 1-(2-fluorophenyl)piperazine, 1-(4-hydroxyphenyl)-piperazine, 1-(4-chlorophenyl)piperazine, 1-(4-trifluoromethylphenyl)piperazine, 1-(4-nitrophenyl)piperazine, 1-(pyrydin-2-yl)piperazine, and 1-(2-furoyl)piperazine.

The reactions with 1-(4-hydroxyphenyl)piperazine, 1-(4-chlorophenyl)piperazine, 1-(4-trifluoromethylphenyl)piperazine, and 1-(2-furoyl)piperazine under the described conditions did not occur. Attempts were made to modify the reaction conditions (classical method), such as heating the reaction mixture for 48 h, using acetonitrile, as a solvent, and extending the reaction time at room temperature to 5 days. The attempts made failed.

4-(3-chlorophenyl)-5-(3-fluorophenyl)-2-[(4-phenylpiperazin-1-yl)methyl]-2,4-dihydro-3*H*-1,2,4-triazole-3-thione (**5**)

White crystals. Yield: 41%, m.p. 132–134 °C. ^1^H NMR (DMSO-*d*_6_) δ (ppm): 2.97–3.01 (m, 4H, 2CH_2_), 3.15–3.19 (m, 4H, 2CH_2_), 5.27 (s, 2H, CH_2_), 6.78 (t, 1H, ArH *J* = 7.6 Hz), 6.94 (d, 2H, ArH, *J* = 8.0 Hz), 7.19–7.22 (m, 4H, ArH), 7.32–7.36 (m, 1H, ArH), 7.42–7.48 (m, 2H, ArH), 7.55 (t, 1H, ArH, *J* = 7.7 Hz), 7.60 (d, 1H, ArH, *J* = 8.2 Hz), 7.71 (s, 1H, ArH). ^13^C NMR (DMSO-*d*_6_) δ (ppm): 48.85, 50.30, 69.44, 115.90, 116.10, 118.20 (d, *J* = 21.0 Hz), 119.46, 125.37, 127.77, 127.83, 128.25, 129.38, 129.55, 130.30, 131.42, 133.78, 136.53, 148.32, 161.18, 162.80, 170.05. Elemental analysis for C_25_H_23_ClFN_5_S. Calculated: C 62.56; H 4.83; N 14.59. Found: C 62.45; H 4.70; N 14.40. HRMS (ESI) calcd. for C_25_H_24_ClFN_5_S [M + H]^+^ 480.14250, found 480.14241.

4-(3-chlorophenyl)-5-(3-fluorophenyl)-2-[4-(2-methoxyphenyl)piperazin-1-yl)methyl]-2,4-dihydro-3*H*-1,2,4-triazole-3-thione (**6**)

White powder. Yield 60%, m.p. 145–147 °C. ^1^H NMR (DMSO-*d*_6_) δ (ppm): 2.98 (s, 8H, 4CH_2_), 3.75 (s, 3H, CH_3_), 5.25 (s, 2H, CH_2_), 6.87–6.95 (m, 4H, ArH), 7.20–7.24 (m, 1H, ArH), 7.33–7.37 (m, 2H, ArH), 7.43–7.49 (m, 2H, ArH), 7.54–7.56 (m, 1H, ArH), 7.59–7.63 (m, 1H, ArH), 7.71–7.73 (m, 1H). ^13^C NMR (DMSO-*d*_6_) δ (ppm): 50.61, 55.60, 69.71, 112.05, 115.97 (d, *J* = 23.8 Hz), 118.21 (d, *J* = 21.0 Hz), 118.51, 121.21, 123.02, 125.36, 127.76, 128.25, 129.54, 130.31, 131.44, 133.80, 136.54, 141.57, 148.33, 152.41, 161.19, 162.82, 170.08. Elemental analysis for C_26_H_25_ClFN_5_OS. Calculated: C 61.23; H 4.94; N 13.73. Found: C 61.35; H 4.80; N 13.60. HRMS (ESI) calcd. for C_26_H_26_ClFN_5_OS [M + H]^+^ 510.15306, found 510.15309.

4-(3-chlorophenyl)-5-(3-fluorophenyl)-2-[4-(2-fluorophenyl)piperazin-1-yl)methyl]-2,4-dihydro-3*H*-1,2,4-triazole-3-thione (**7**)

White crystals. Yield: 62%, m.p. 137–139 °C. ^1^H NMR (DMSO-*d*_6_) δ (ppm): 3.01–3.04 (m, 8H, 4CH_2_), 5.26 (s, 2H, CH_2_), 6.95–6.99 (m, 1H, ArH), 7.04–7.07 (m, 1H, ArH), 7.10–7.14 (m, 2H, ArH), 7.19–7.24 (m, 2H, ArH), 7.33–7.36 (m, 1H, ArH), 7.43–7.49 (m, 2H, ArH), 7.55 (t, 1H, ArH *J* = 8.0 Hz), 7.59–7.61 (m, 1H, ArH), 7.72 (t, 1H, ArH *J* = 8.0 Hz). ^13^C NMR (DMSO-*d*_6_) δ (ppm): 50.33, 50.64, 69.52, 115.96 (d, *J* = 23.8 Hz), 116.39 (d, *J* = 20.7 Hz), 118.20 (d, *J* = 20.8 Hz), 119.84, 122.97, 125.35, 126.57, 128.26, 129.54, 130.31, 131.43, 133.79, 136.54, 140.37, 148.36, 154.64, 156.36, 161.26, 162.81, 170.05. Elemental analysis for C_25_H_22_ClF_2_N_5_S. Calculated: C 60.30; H 4.45; N 14.06. Found: C 60.35; H 4.30; N 13.95. HRMS (ESI) calcd. for C_25_H_23_ClF_2_N_5_S [M + H]^+^ 498.13308, found 498.13313.

4-(3-chlorophenyl)-5-(3-fluorophenyl)-2-[4-(4-nitrophenyl)piperazin-1-yl)methyl]-2,4-dihydro-3*H*-1,2,4-triazole-3-thione (**8**)

Red crystals. Yield 44%, m.p. 235–237 °C. ^1^H NMR (DMSO-*d*_6_) δ (ppm): 2.96–2.98 (m, 4H, 2CH_2_), 3.51–3.53 (m, 4H, 2CH_2_), 5.27 (s, 2H, CH_2_), 7.04 (d, 2H, CH arom, *J* = 9.1 Hz), 7.18 (t, 2H, ArH, *J* = 8.0 Hz), 7.33 (t, 1H, ArH, *J* = 8.6 Hz), 7.40–7.47 (m, 2H, ArH), 7.54 (t, 1H, ArH, *J* = 8.0 Hz), 7.58–7.60 (m, 1H, ArH), 7.68 (s, 1H, ArH), 8.04 (d, 2H, ArH *J* = 9.0 Hz). ^13^C NMR (DMSO-*d*_6_) δ (ppm): 46.24, 49.97, 69.28, 115.96 (d, *J* = 24.0 Hz), 118.21 (d, *J* = 20.6 Hz), 125.34, 126.21, 127.76, 128.21, 129.49, 130.31, 133.78, 136.48, 137.28, 148,38, 155.13, 161.16, 162.78, 170.05. Elemental analysis for C_25_H_22_ClFN_6_O_2_S. Calculated: C 57.19; H 4.22; N 16.01. Found: C 57.15; H 4.10; N 15.90. HRMS (ESI) calcd. for C_25_H_22_ClFN_6_O_2_S [M + H]^+^ 525.12758, found 525.12751.

4-(3-chlorophenyl)-5-(3-fluorophenyl)-2-[4-(pyrydin-2-yl)piperazin-1-ylmethyl]-2,4-dihydro-3*H*-1,2,4-triazole-3-thione (**9**)

White crystals. Yield: 32%, m.p. 165–167 °C. ^1^H NMR (DMSO-*d*_6_) δ (ppm): 2.84–2.94 (m, 4H, 2CH_2_), 3.76–3.83 (m, 4H, 2CH_2_), 5.26 (s, 2H, CH_2_), 6.60–6.63 (m, 1H, ArH), 7.15–7.20 (m, 2H, ArH), 7.31–7.34 (m, 1H, ArH), 7.40–7.60 (m, 5H, ArH), 7.69 (s, 1H, ArH), 8.35 (s, 2H, ArH). ^13^C NMR (DMSO-*d*_6_) δ (ppm): 43.59, 50.22, 69.64, 110.52, 115.95 (d, *J* = 23.9 Hz), 118.17 (d, *J* = 20.7 Hz), 125.35, 127.74, 128.25, 129.52, 130.29, 131.40, 133.75, 136.53, 148.33, 158.40, 161.15, 161.54, 162.78, 170.02. Elemental analysis for C_24_H_22_ClFN_6_S. Calculated: C 59.93; H 4.61; N 17.47. Found: C 59.65; H 4.50; N 17.40. HRMS (ESI) calcd. for C_24_H_23_ClFN_6_S [M + H]^+^ 481.13775, found 481.13773.

### 3.2. Antibacterial and Antifungal Activity

The obtained compounds were screened for their potential antimicrobial activity. A broad panel of reference strains from the American Type Culture Collection (ATCC), including bacteria (Gram-positive: *Staphylococcus epidermidis* ATCC 12228, *Staphylococcus aureus* ATCC 43300, *Micrococcus luteus* ATCC 10240, *Bacillus cereus* ATCC 10876, and *Bacillus subtilis* ATCC 6633; Gram-negative: *Escherichia coli* ATCC 3521, *Pseudomonas aeruginosa* ATCC 27853, *Klebsiella pneumoniae* ATCC 13883, and *Proteus mirabilis* ATCC 12453) and yeasts (*Candida glabrata* ATCC 15126, *Candida krusei* ATCC 14243, and *Candida parapsilosis* ATCC 22019) came from the collection of the Department of Pharmaceutical Microbiology of the Medical University of Lublin, Poland.

#### 3.2.1. Minimum Inhibitory Concentration (MIC) Assay

Antimicrobial activity of new piperazine derivatives was determined using the microdilution broth method according to both the European Committee on Antimicrobial Susceptibility Testing (EUCAST) and Clinical and Laboratory Standards Institute (CLSI) procedures [41,42], as previously described [32,43]. Suspensions of the test bacteria were then prepared. For this purpose, 1–2 colonies were collected with a moistened sterile swab into a sterile tube containing 2 mL of sterile 0.85% NaCl solution (Biomerieux, Craponne, France). The prepared suspension was brought to a density of 0.5 on the McFarland scale (5 × 10^5^ CFU/mL; CFU—colony forming units). Fresh 24 h cultures were suspended in sterile 0.85% NaCl to obtain 0.5 McFarland density, then diluted 100-fold in sterile Mueller–Hinton Broth (MHB, Biomaxima, Lublin, Poland) or Mueller–Hinton Broth with 2% glucose (MHB+2%glc, Biomaxima, Lublin, Poland) media to obtain a final density of 1.5 × 10^6^ colony-forming units (CFU)/mL of bacteria and yeasts, respectively. Stock solutions of newly synthesized compounds at initial 50 mg/mL concentration were prepared by dissolution in 1 mL of dimethyl sulfoxide (DMSO).

The first wells of the 96-well plate (Medlab, Lublin, Poland) were filled with 200 µL of compounds tested at a concentration of 1000 µg/mL using an automated pipette, and the remaining wells were filled with 100 µL of sterile liquid medium under aseptic conditions. The contents of the wells were mixed thoroughly and serial dilutions were made using a multichannel automatic pipette. This resulted in two-fold decreasing concentrations of compounds in the range 0.49–1000 µg/mL.

Concurrently with the determination of the activity of the tested compounds against the model bacterial and fungal strains, the following were performed: (a) a viability control of the test bacterial and fungal strains by adding 2 µL of the diluted suspension to three wells of pure medium for each strain, (b) a pure medium control by adding 100 µL of sterile medium to the wells of the titer plate without the addition of the test compound, (c) a test extract control with the same dilution series without the addition of the microbial suspension, and (d) a positive control with the commercially available drugs—ciprofloxacin and posaconazole (Sigma, St. Louis, MO, USA). Each experiment was repeated in triplicate and representative data are presented.

Microplates prepared in this way were incubated for 18 ± 2 h at 35 ± 1 °C under aerobic conditions. After this time, microbial growth in individual wells of the plate was read using a Bio-Tek Elx800 spectrophotometric reader (Biokom, Janki, Poland) at 570 nm and macroscopic observation of visible turbidity. Measurements were then processed using the Gen5 ver. 3.03.14 (Biokom, Poland). Bacterial growth was indicated by visible turbidity in the wells of the plate. The effect of the tested extract against bacterial or fungal strains was assessed on the basis of the minimum inhibitory concentration (MIC) values.

#### 3.2.2. Minimum Bactericidal (MBC) and Fungicidal (MFC) Concentration Assay

The lowest concentration of newly synthesized compounds at which no growth of 99.9% of the bacteria was observed was considered the minimum bactericidal or fungicidal concentration (MBC/MFC) value.

To determine those parameters, 5 µL of the culture was aseptically transferred from the well in the MIC assay (the one presenting the MIC value and two wells above it) onto a sterile Mueller–Hinton agar and Mueller–Hinton agar + 2% glucose for bacteria and yeasts, respectively. The prepared agar media were then incubated at 35 ± 2 °C for 24 h under aerobic conditions. After incubation, the lowest concentration of the compounds tested caused complete inhibition of the growth of selected strains of bacteria and fungi.

On the basis of the results obtained and the MBC/MIC ratio, the bactericidal or fungicidal activity (MBC/MIC or MFC/MIC ≤ 4) or bacterio/fungistatic activity (MBC/MIC or MFC/MIC > 4) of the tested compounds against the tested microorganisms was determined.

## 4. Conclusions

In summary, the group of newly synthesized piperazine derivatives obtained in Mannich reactions are promising compounds with both antibacterial and antifungal activity. Significant activity was observed against either Gram-positive bacteria, mainly staphylococci (**8**–**9**), *Micrococcus* spp., and *Bacillus* spp. (**5**–**7**), or Gram-negative bacteria, including bacteria from the *Enterobacteriaceae* family (**8**). In addition, all compounds tested showed high fungistatic activity against *Candida parapsilosis*, with MICs ranging from 0.49 µg/mL (**8**) to 0.98 µg/mL (**9**) and 62.5 µg/mL (**5**–**7**). Based on the in silico research results, the tested compounds are expected to have good oral bioavailability and membrane permeability, with generally high absorption in the gastrointestinal tract.

To conclude, the results obtained confirm the multi-directional antimicrobial activity of the new piperazine derivatives and show how structural substitution affects the activity of this class of compounds against different microbes. The results obtained are expected to provide valuable data for further exploring this interesting group. Pharmacological research aimed at finding new interesting biologically active compounds is continuing on the library of compounds obtained.

## Figures and Tables

**Table 1:** In vitro antimicrobial activity of new piperazine derivatives.

Compound		5			6			7			8			9			Positive Control Drug	
																	CIP	
Microorganism		MIC	MBC	MBC/ MIC	MIC	MBC	MBC/ MIC	MIC	MBC	MBC/MIC	MIC	MBC	MBC/MIC	MIC	MBC	MBC/MIC	MIC	MBC
Gram-positive bacteria	*S. epidermidis* ATCC 12228	500	>	-	1000	>	-	>	n.d.	-	125	500	4	250	500	2	1.48	2
	*S. aureus* ATCC 25923	n.d.	n.d.	-	>	n.d.	-	>	n.d.	-	125	250	2	250	500	2	2.96	4
	*M. luteus* ATCC 10240	125	125	1	125	500	4	125	250	2	31.25	500	16	62.50	250	4	5.88	8
	*B. subtilis* ATCC 6633	250	500	2	1000	1000	1	250	250	1	15.63	500	32	125	250	2	0.09	0.15
	*B. cereus* ATCC 10876	500	1000	2	1000	1000	1	500	1000	2	125	500	4	125	1000	4	0.36	0.5
Gram-negative bacteria	*E. coli* ATCC 25922	>	n.d.	-	>	n.d.	-	>	n.d.	-	250	500	2	>	n.d.	-	0.024	0.5
	*P. aeruginosa* ATCC 27853	>	n.d.	-	>	n.d.	-	>	n.d.	-	>	n.d.	-	>	n.d.	-	0.72	1
	*K. pneumoniae* ATCC 13883	>	n.d.	-	1000	n.d.	-	>	n.d.	-	125	125	1	>	n.d.	-	0.36	0.5
	*P. mirabilis* ATCC 12453	>	n.d.	-	>	n.d.	-	>	n.d.	-	>	500	-	>	n.d.	-	0.045	0.15
		**MIC**	**MFC**	**MFC/MIC**	**MIC**	**MFC**	**MFC/MIC**	**MIC**	**MFC**	**MFC/MIC**	**MIC**	**MFC**	**MFC/MIC**	**MIC**	**MFC**	**MFC/MIC**	**POS**	
																	**MIC**	**MFC**
yeasts	*C. krusei* ATCC 14243	>	n.d.	-	>	n.d.	-	>	n.d.	-	>	n.d.	-	>	n.d.	-	0.125	1
	*C. glabrata* ATCC 15126	>	n.d.	-	>	n.d.	-	>	n.d.	-	>	n.d.	-	>	n.d.	-	0.244	16
	*C. parapsilosis* ATCC 22019	62.5	1000	16	62.5	>1000	-	62.5	1000	16	0.49	>1000	-	0.98	1000	1020	0.016	1

**Abbreviations**: MIC—minimal inhibitory concentration [µg/mL], MBC—minimal bactericidal concentration [µg/mL], MFC—minimal fungicidal concentration [µg/mL], CIP—ciprofloxacin; POS—posaconazole; n.d.—not detected; green-stained table cells—bactericidal effect resulting from MBC/MIC ratio < 4.

**Table 2:** Physicochemical properties of new compounds obtained using the SWISSADME server.

Lipinski’s Rule of Five (Ro5)					
Compound	H-Bond Acceptors	H-Bond Donors	MW [g/mol]	Log P (MLOGP)	Violations
**5**	3	0	480.00	4.77	1
**6**	4	0	510.03	4.56	2
**7**	4	0	497.99	5.13	1
**8**	5	0	525.00	3.78	1
**9**	4	0	480.99	4.16	1

**Table 3:** Predicted ADME parameters of new compounds obtained using the SWISSADME server.

Pharmacokinetics Parameters				
Compound	GI Absorption	BBB Permeant	P-gp Substrate	Water Solubility
**5**	High	Yes	Yes	Poorly soluble
**6**	High	No	Yes	Poorly soluble
**7**	High	No	Yes	Poorly soluble
**8**	Low	No	No	Poorly soluble
**9**	High	No	Yes	Poorly soluble

**Table 4:** Predicted drug-likeness parameters of new compounds obtained using the SWISSADME server.

Pharmacokinetics Parameters				
Compound	GI Absorption	BBB Permeant	P-gp Substrate	Water Solubility
**5**	High	Yes	Yes	Poorly soluble
**6**	High	No	Yes	Poorly soluble
**7**	High	No	Yes	Poorly soluble
**8**	Low	No	No	Poorly soluble
**9**	High	No	Yes	Poorly soluble

## Data Availability

The details of the data supporting the reported results in this research were included in the paper and Supplementary Materials.

## Supplementary Materials

The following supporting information can be downloaded at: https://www.mdpi.com/article/10.3390/molecules28145562/s1, Figures S1–S5: ^1^H NMR spectra for titled compounds, Figures S6–S10: ^13^C NMR spectra for titled compounds.

## Sample Availability

Samples of the compounds are available from the authors.
