# Supplementary material for: Synthesis and Antimicrobial Activity of New Mannich Bases with Piperazine Moiety
Source: Molecules. 2023 Jul 21;28(14):5562. doi: 10.3390/molecules28145562 (PMC10384309; doi:10.3390/molecules28145562)
Supplement: Supplementary file 1 [file molecules-28-05562-s001.zip › molecules-2498957-supplementary.pdf]

Supplementary Information

Synthesis and antimicrobial activity of new Mannich bases with piperazine moiety

Sara Janowska[1*], Sylwia Andrzejczuk[2], Piotr Gawryś[3] and Monika Wujec[1*]

[1]Department of Organic Chemistry, Faculty of Pharmacy, Medical University of Lublin, 4a Chodzki Street, 20-093 Lublin, Poland
[2]Department of Pharmaceutical Microbiology, Faculty of Pharmacy, Medical University of Lublin, 1 Chodzki Street, 20-093 Lublin, Poland
[3]Students Research Group at the Department of Organic Chemistry

[*]corresponding authors: monika.wujec@umlub.pl, sara.janowska@umlub.pl

Table of contents:

1. ^{1}H NMR spectra ……………………………………..……………………………………2
2. ^{13}C NMR spectra ……….…………………………………………………………......6

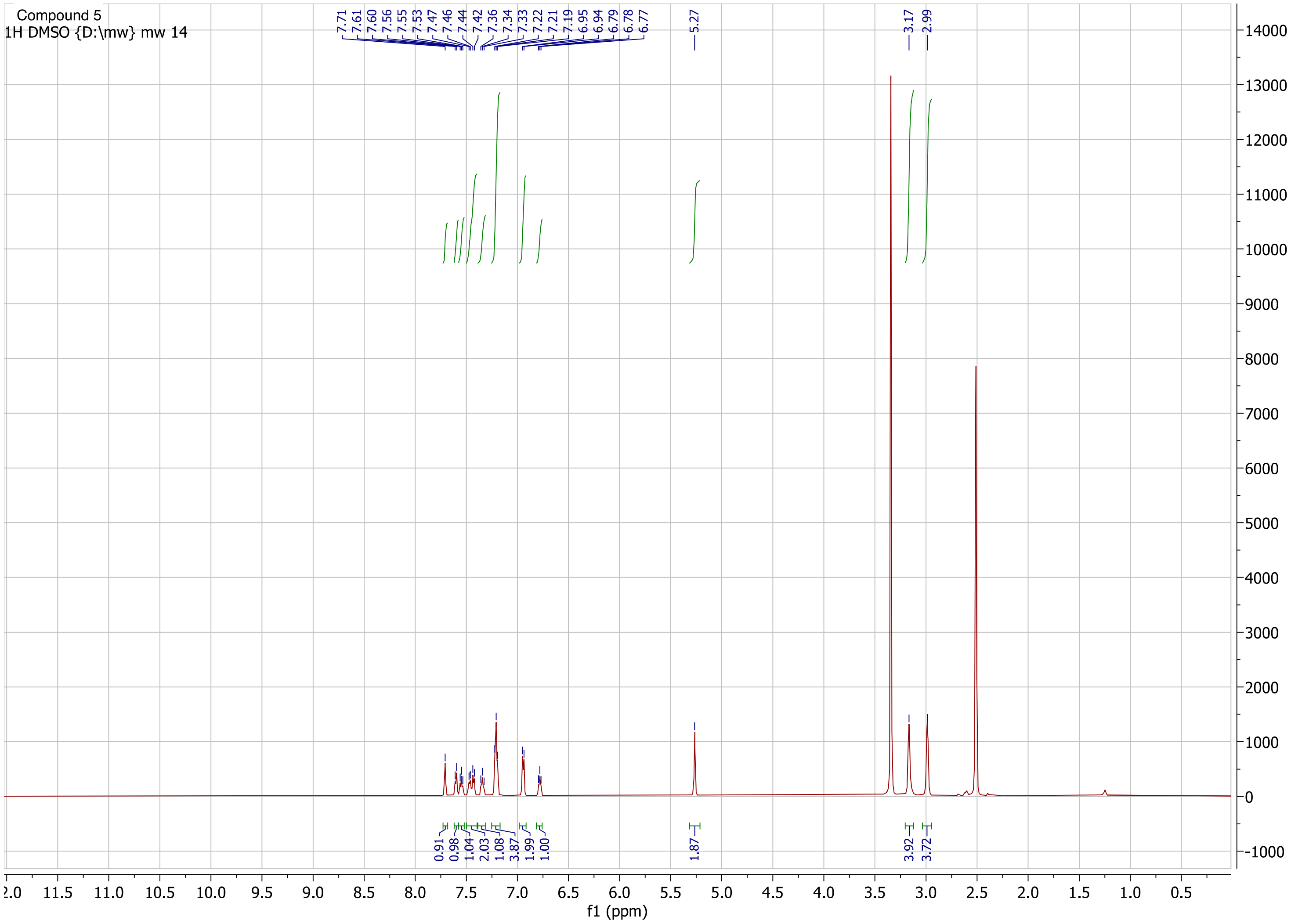

Figure S1. The ^{1}H NMR of compound 5.

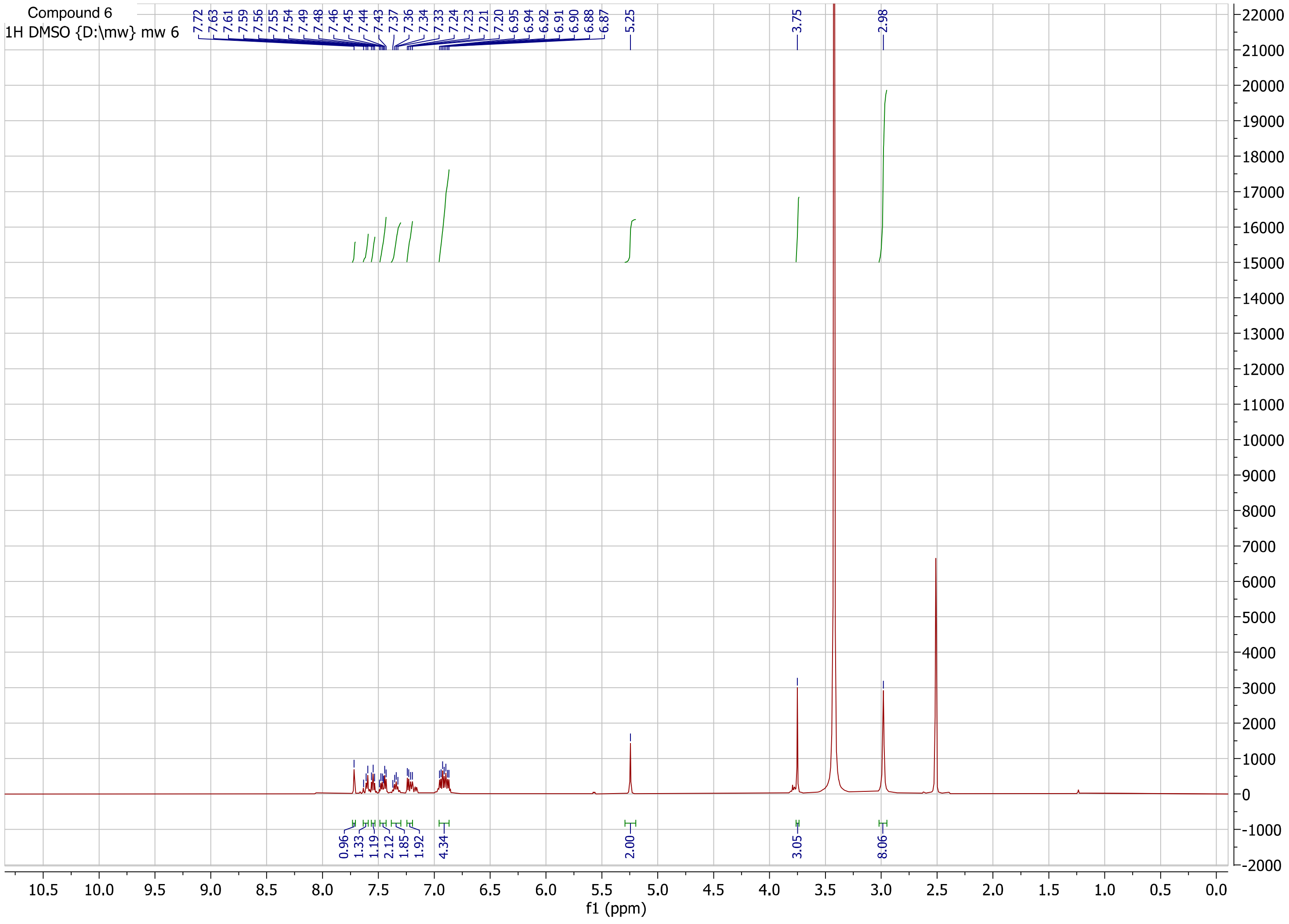

Figure S2. The ^{1}H NMR of compound 6.

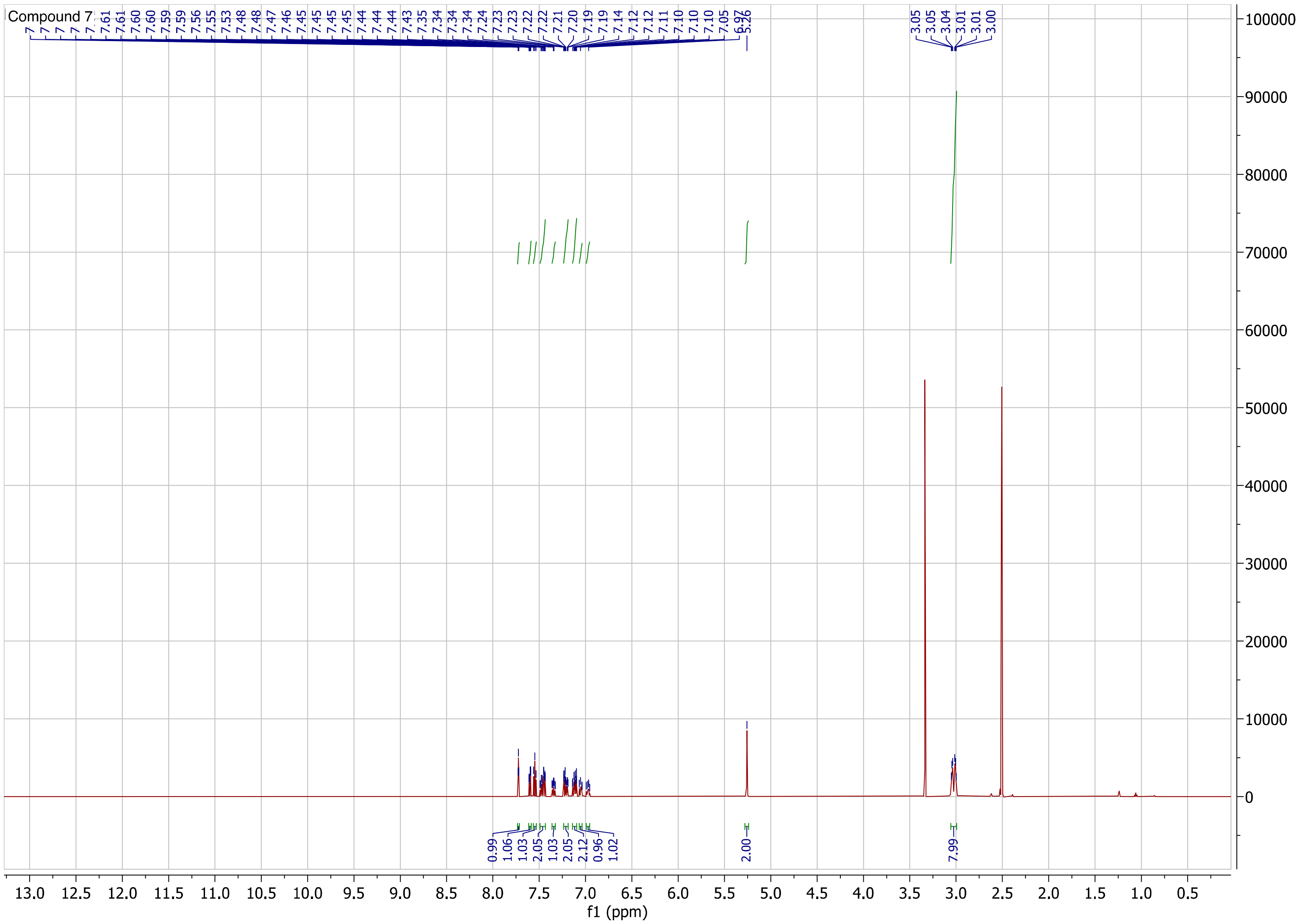

Figure S3. The ^{1}H NMR of compound 7.

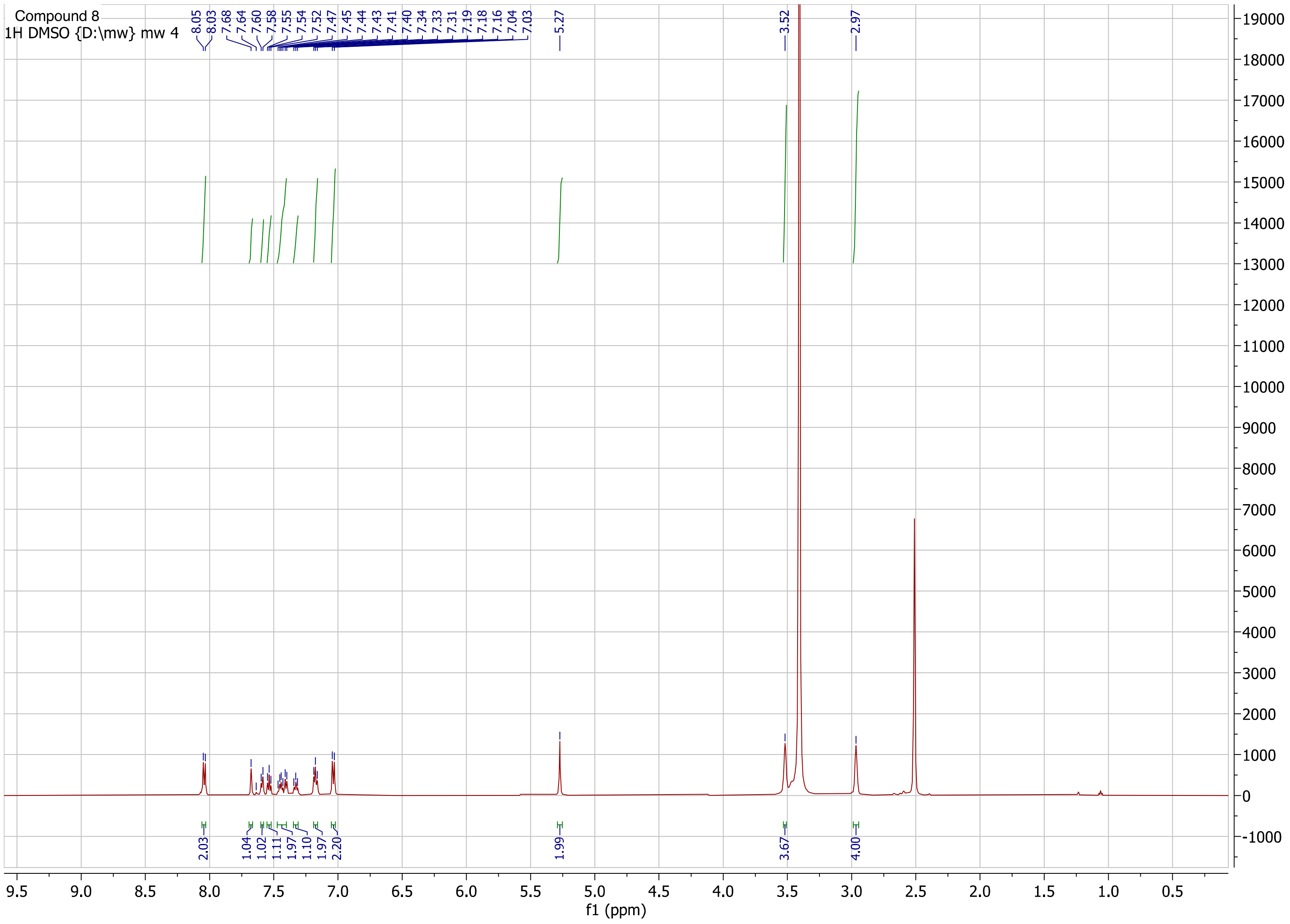

Figure S4. The ^{1}H NMR of compound 8.

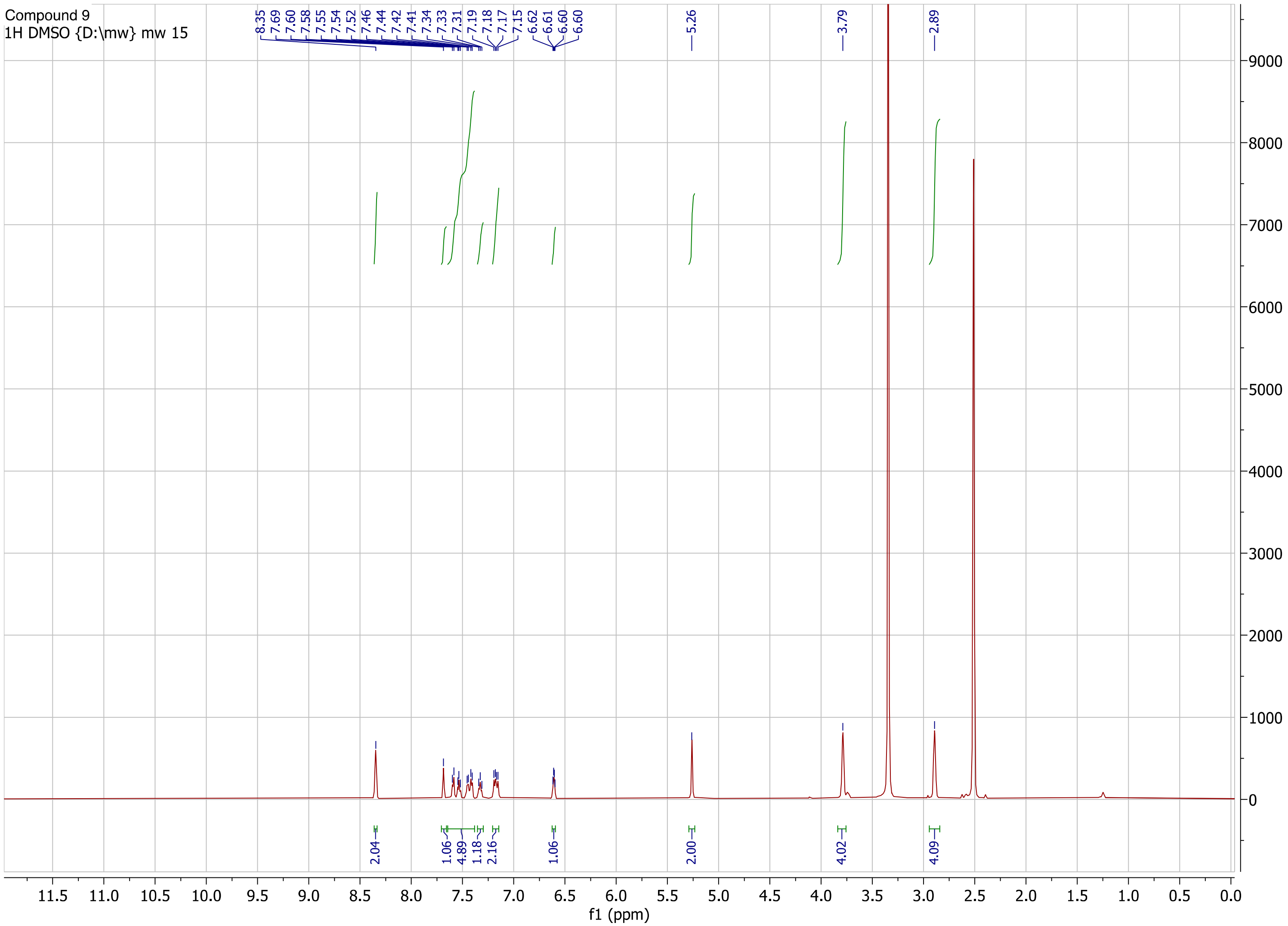

Figure S5. The ^{1}H NMR of compound 9.

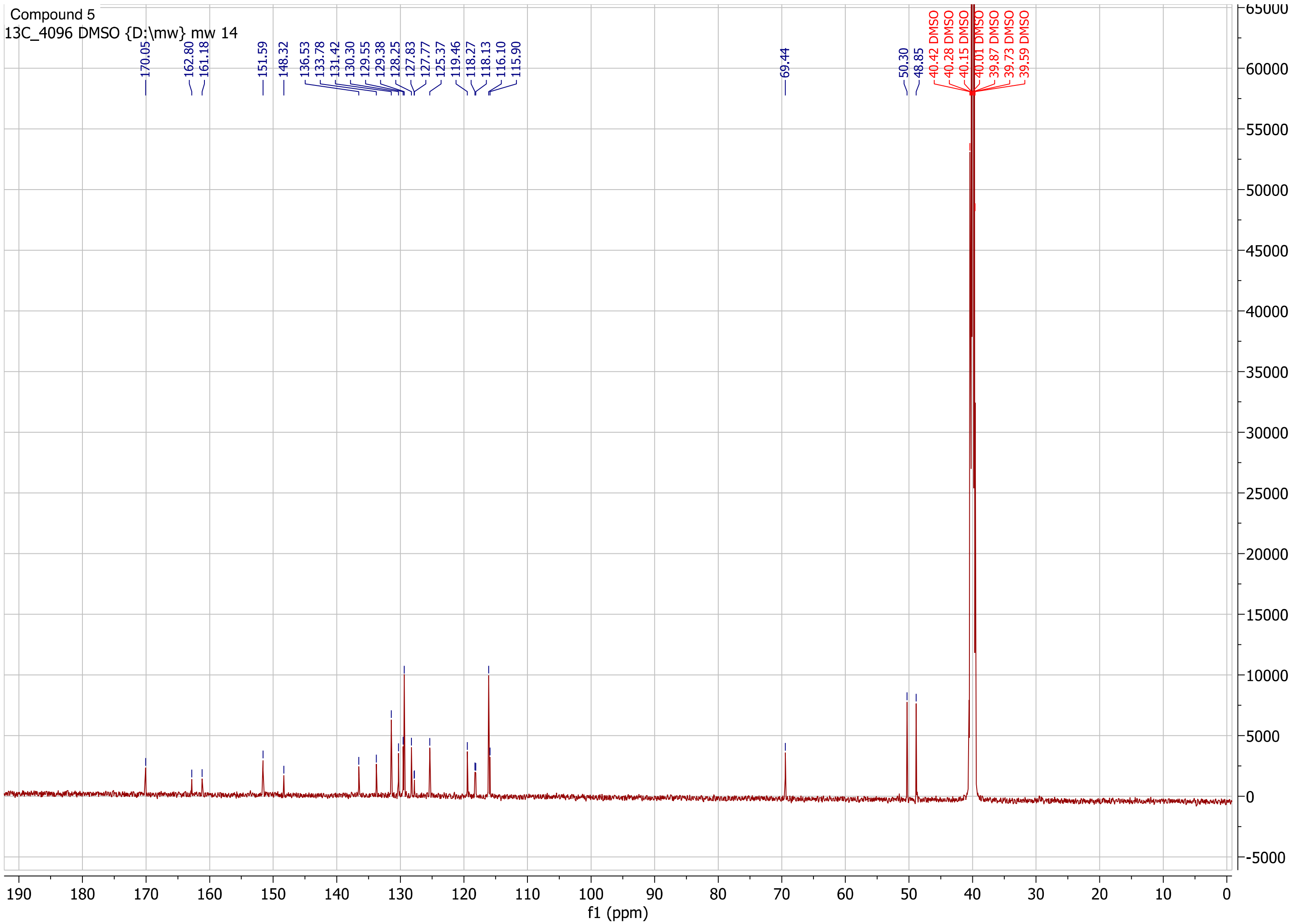

Figure S6. The ^{13}C NMR of compound 5.

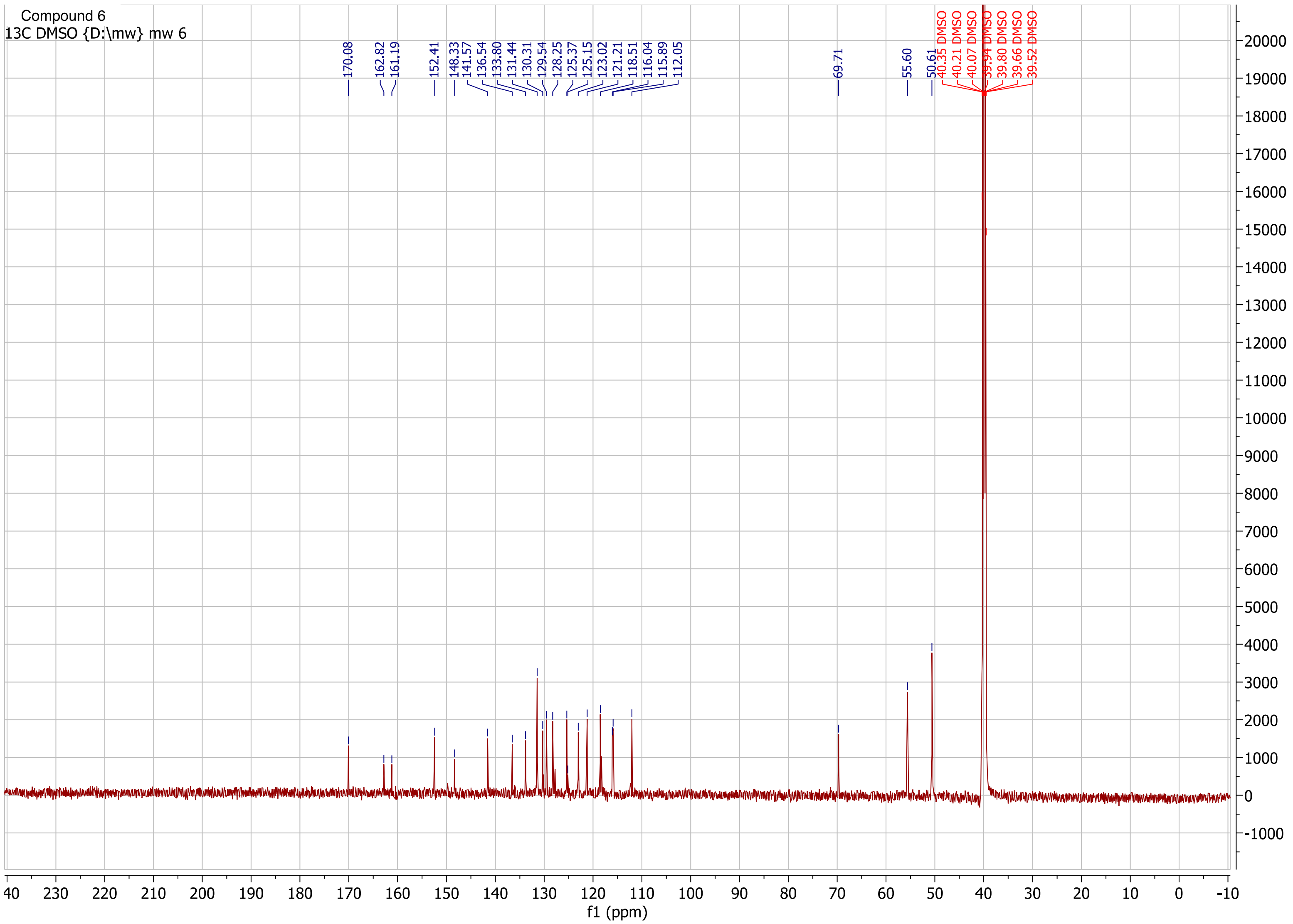

Figure S7. The ^{13}C NMR of compound 6.

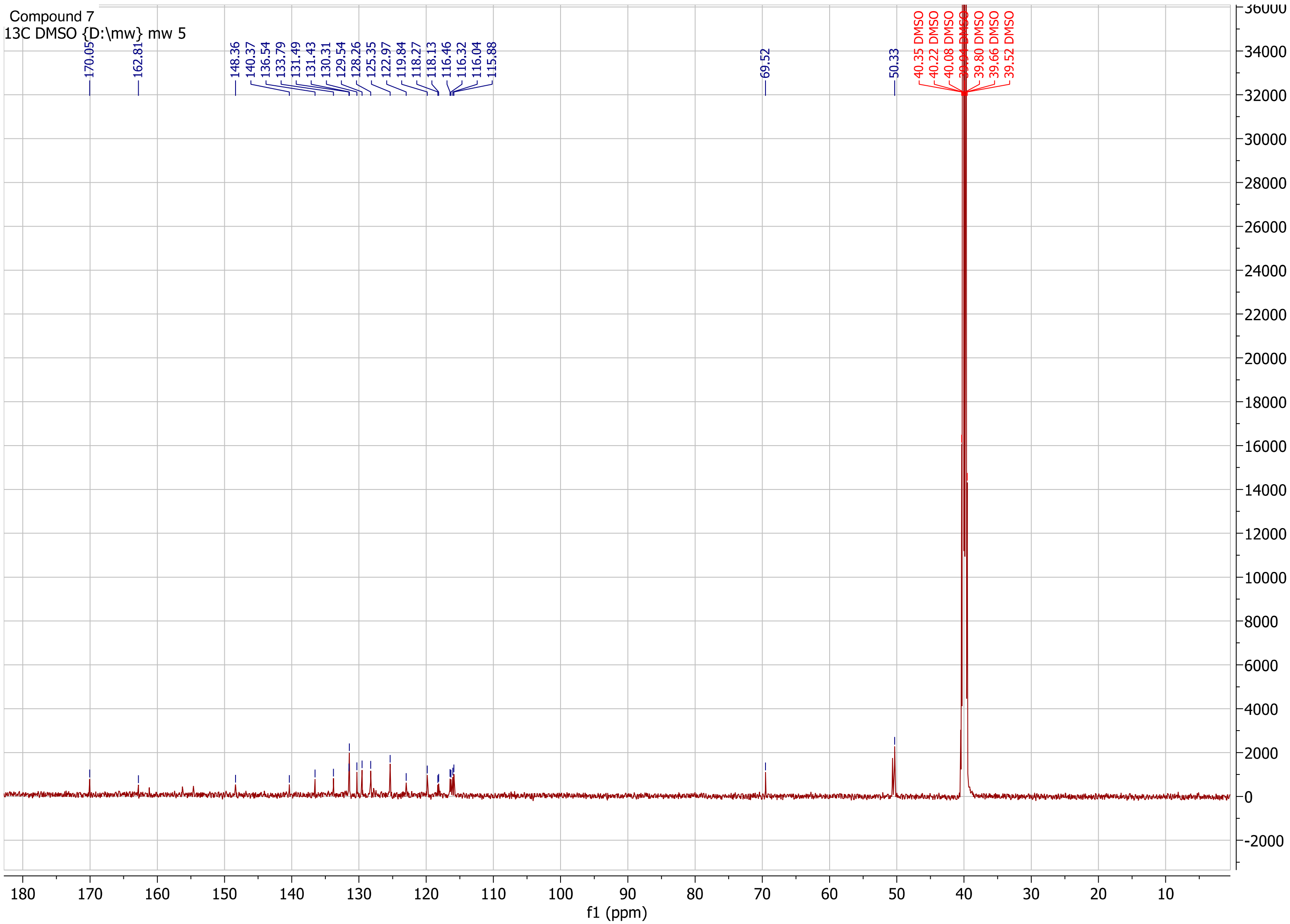

Figure S8. The ^{13}C NMR of compound 7.

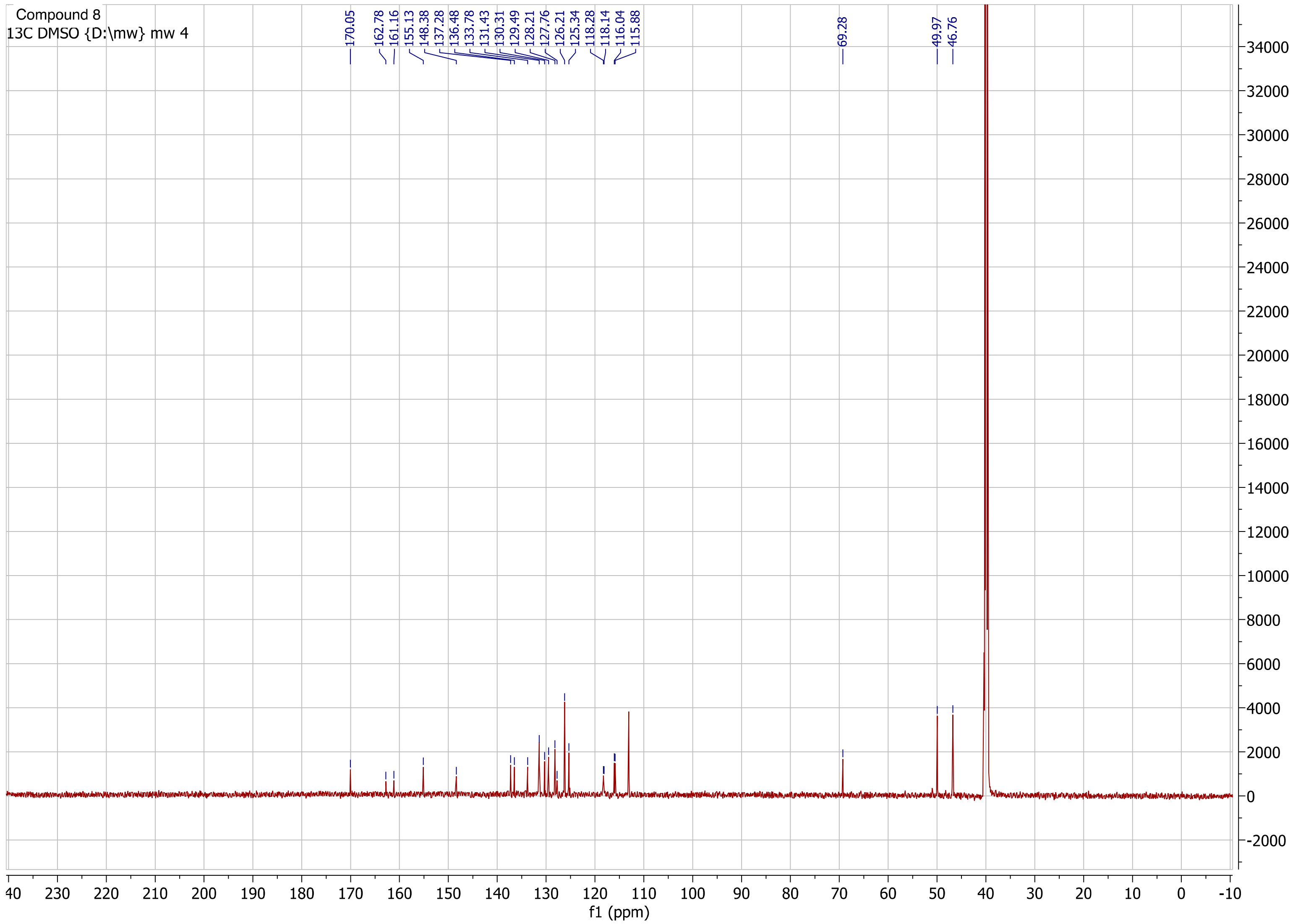

Figure S9. The ^{13}C NMR of compound 8.

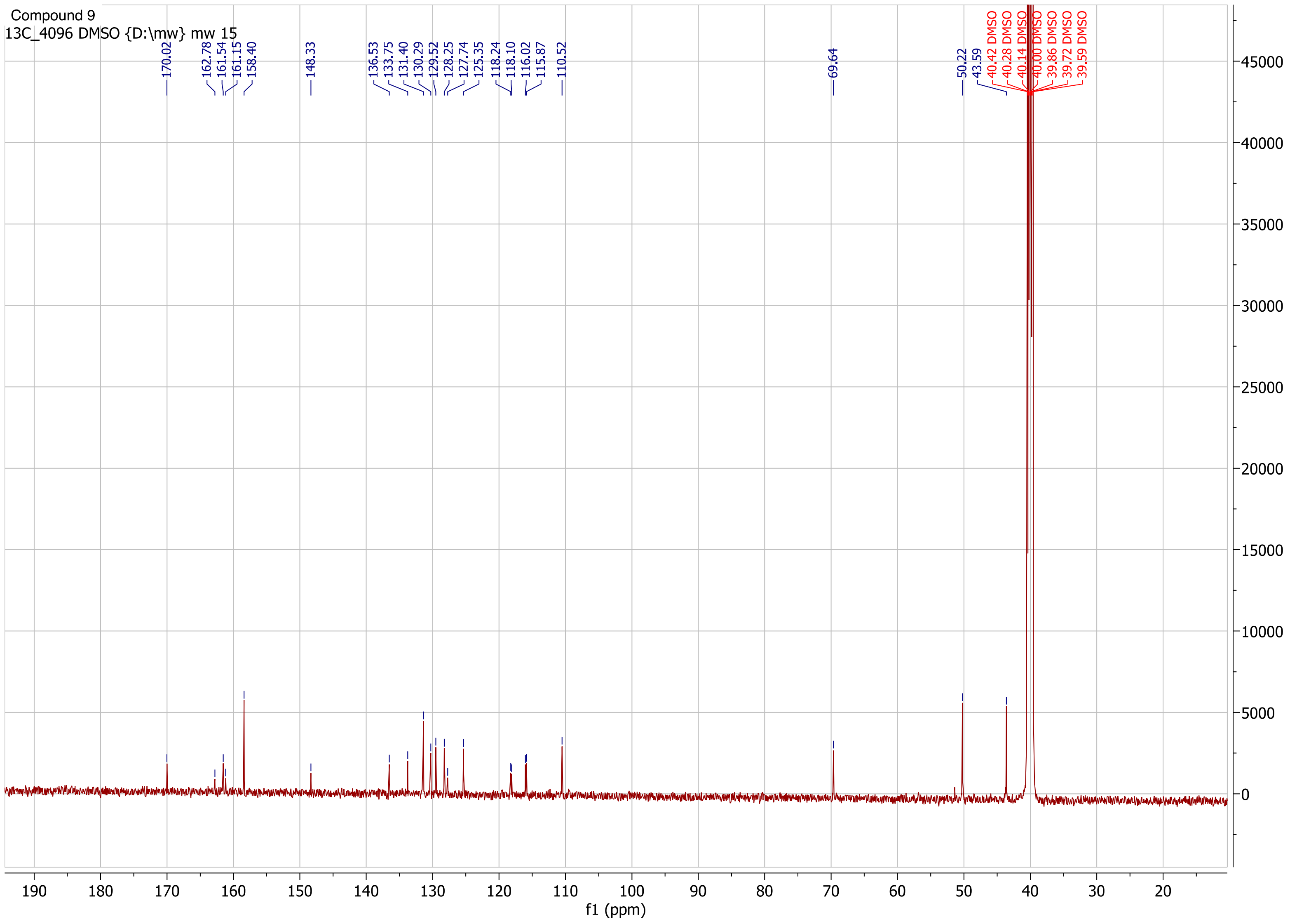

Figure S10. The ^{13}C NMR of compound 9.
